# Towards reliable hyperspectral imaging biomarkers of CT26 murine tumor model

**DOI:** 10.1016/j.heliyon.2024.e39816

**Published:** 2024-10-26

**Authors:** Tadej Tomanic, Jost Stergar, Tim Bozic, Bostjan Markelc, Simona Kranjc Brezar, Gregor Sersa, Matija Milanic

**Affiliations:** a Faculty of Mathematics and Physics, University of Ljubljana, 1000 Ljubljana, Slovenia; b Jozef Stefan Institute, 1000 Ljubljana, Slovenia; c Department of Experimental Oncology, Institute of Oncology Ljubljana, 1000 Ljubljana, Slovenia; d Faculty of Medicine, University of Ljubljana, 1000 Ljubljana, Slovenia; e Faculty of Health Sciences, University of Ljubljana, 1000 Ljubljana, Slovenia

**Keywords:** Biomarkers, Hyperspectral imaging, Machine learning, Murine models, Tumors

## Abstract

The non-invasive monitoring of tumor growth can offer invaluable diagnostic insights and enhance our understanding of tumors and their microenvironment. Integrating hyperspectral imaging (HSI) with three-dimensional optical profilometry (3D OP) makes contactless and non-invasive tumor diagnosis possible by utilizing the inherent tissue contrast provided by visible (VIS) and near-infrared (NIR) light. Consequently, valuable information regarding tumors and healthy tissues can be extracted from the acquired hyperspectral images. Until now, very few methods have been used to monitor tumor models *in vivo* daily and non-invasively. In this research, we conducted a 14-day study monitoring BALB/c mice with subcutaneously grown CT26 murine colon carcinomas *in vivo*, commencing on the day of tumor cell injection. We extracted physiological properties such as total hemoglobin (THB) and tissue oxygenation (${\mathrm{StO}}_{2}$) using the inverse adding-doubling (IAD) algorithm and manually segmented the tissues. We then selected the ten most relevant features describing tumors using the Max-Relevance Min-Redundancy (MRMR) algorithm and utilized 30 classic and advanced machine learning (ML) algorithms to discriminate tumors from healthy tissues. Finally, we tested the robustness of feature selection and model performance by smoothing tissue parameter maps extracted by IAD with a variable kernel and omitting selected training data. We could discriminate CT26 tumor models from surrounding healthy tissues with an area under the curve (AUC) of up to 1 for models based on the gradient boosting method, linear discriminant analysis, and random forests. Our findings help pave the way for precise and robust imaging biomarkers that could aid tumor diagnosis and advance clinical practice.

## 1 Introduction

Cancer is a significant public health problem and one of the major causes of death globally [1], [2], [3]. According to the World Health Organization (WHO), cancer accounted for approximately 10 million deaths worldwide in 2020. Early cancer diagnosis is critical for patients to expand their treatment options and improve survival rates [3].

Skin and subcutaneous lesions, such as skin cancer, are among the most common malignancies in adult humans. For example, the incidence of malignant melanoma is expected to increase by 0.6% per year among adults over 50 years [4]. Such an increase significantly strains the healthcare infrastructure, creating a demand for rapid, precise, cost-effective diagnostic instruments. In the current clinical setting, histology is the gold standard for diagnosing skin lesions, but it is highly invasive, time-consuming, and examines small parts of tumors. On the other hand, optical methods are usually non-invasive, fast, can image large areas of tissue, and are sensitive to intrinsic changes in light absorption and scattering in tumors, potentially discriminating them from healthy tissues [5], [6].

**Figure 1:**
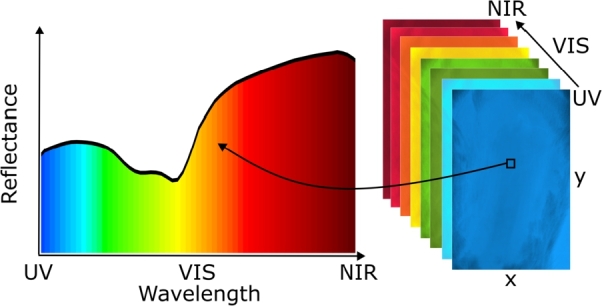
A representation of a hyperspectral image and a skin reflectance spectrum.

Hyperspectral imaging (HSI) is an emerging optical method combining imaging and spectroscopy that is contactless, non-invasive, and affordable [7], [8], [9]. HSI captures spatial and spectral data of the examined tissue sample within a hyperspectral image, commonly in the ultraviolet (UV), visible (VIS), and near-infrared (NIR) spectral ranges. The imaging systems can operate in various modes, capturing spatially-resolved reflectance or transmittance spectra of the sample. As a result, the acquired hyperspectral images contain spectral signatures of a tissue sample for each pixel within the image (see Fig. 1). These signatures are fingerprints of the substances constituting the tissue [10]. The prominence of spectral signatures is related to the volume fractions or concentrations of tissue absorbers, also called chromophores. The main chromophores in the skin are melanin and hemoglobin [6], whose concentrations can be altered in tumors due to their hallmarks, including changes in metabolism and the formation of new blood vessels (angiogenesis) [11], [12]. Consequently, spectral signatures of tumors are expected to differ from healthy tissues, allowing for efficient tumor diagnosis using HSI.

In recent years, multiple studies have investigated the application of HSI to detect skin and subcutaneous lesions [13], [14], [15], [16], [17]. In one of the few preclinical applications, Sorg et al. [18] used 4T1 mouse mammary carcinomas grown in dorsal window chambers (DWCs) to study hemoglobin saturation and tumor hypoxia development *in vivo*. Although the imaging is non-invasive, the surgical implantation of titanium window chambers is highly invasive.

The majority of other studies were performed in humans. Nagaoka et al. [19] proposed an objective melanoma discrimination index based on hyperspectral data that can detect melanomas with a sensitivity and specificity of 90% and 84%, respectively. Zheludev et al. [20] delineated malignant skin tumors based on spectral signatures by applying the framelet transform and dimensionality reduction and utilizing different machine learning (ML) algorithms. Zherdeva et al. [21] have shown that it is possible to discriminate malignant melanoma (MM) from pigmented nevi (PN) in the *ex vivo* skin tissues based on differences in optical density (OD).

Similarly, they showed that HSI could differentiate between skin cancer and healthy skin based on spectral features and principal component analysis (PCA) with both sensitivity and specificity of around 91% in an *in vivo* study [22]. Recently, Aloupogianni et al. [10] studied the effects of dimension reduction of hyperspectral images in skin gross pathology. Their *ex vivo* skin samples showed that random forest importance (RFI) performed best during classification (Jaccardi coefficient of 46.91), but all their methods suffered from low sensitivity and generalization. Also, Hosking et al. [23] achieved a high sensitivity of 100% but extremely low specificity of 36% in detecting melanoma using multiple ML classification algorithms. Leon et al. [24] reported a sensitivity and specificity of 88% and 100% for discriminating malignant and benign pigmented skin lesions (PSL). Lastly, Calin et al. [25] utilized unsupervised anomaly detection algorithms to detect basal cell carcinoma (BCC) and achieved a maximum AUC of 0.86.

Moreover, Neittaanmäki-Perttu et al. [26] showed that HSI could detect subclinical borders of lentigo maligna (LM) and lentigo maligna melanoma (LMM) in more than 50% of cases using the spectral unmixing technique. The same approach was used in another study where the delineation of ill-defined BCC was more accurate than conventional clinical evaluation in 75% of cases [27]. More recently, the same group used a 3D convolutional neural network (CNN) to classify pigmented BCCs from melanocytic tumors and achieved specificity and sensitivity up to 100% [28]. Furthermore, Hirano et al. [29] discriminated melanoma from non-melanoma lesions using a pre-trained GoogLeNet with a sensitivity of 72% and specificity of 81%. Kato et al. [30] achieved similar results using GoogLeNet, with sensitivity and specificity of 80% and 82%, respectively. Using CNN, Lindholm et al. achieved a sensitivity and specificity of 87% and 93%, respectively [31]. Other applications of HSI to detect skin cancer have used tumor cells [32] and histology slides [33], [34].

What is more, three-dimensional (3D) optical profilometry (OP) is a technique capable of measuring the 3D shape of an object of interest [35]. Norhaimi et al. [36], [37] and Meza et al. [38] showed that OP could be used for shape-based breast cancer detection in breast phantoms. Also, Via et al. [39] demonstrated that OP is advantageous for real-time non-invasive localization of intraocular tumors.

In this study, we monitored CT26 murine colon carcinomas on a daily basis from the day of tumor cell injection to a maximum volume of 200 mm^3^. As CT26 tumors were grown in the subcutaneous layer of the skin, they can be considered subcutaneous lesions, so we acquired images using a custom-built HSI system integrated with 3D OP suitable for imaging skin and subcutaneous lesions. Our main goal was to monitor tumor progression continuously to detect and understand day-to-day changes in tumor physiology and morphology. Our second goal was to identify the most relevant features describing tumor characteristics, focusing on easy interpretability for preclinical and clinical applications. Another goal was to discriminate CT26 tumors from neighboring healthy tissues using classic and advanced ML techniques to aid tumor diagnosis. Our final goal was to test the robustness of the proposed methods and pave the way toward reliable hyperspectral imaging biomarkers in humans.

## 2 Materials and methods

### 2.1 Imaging system

**Figure 2:**
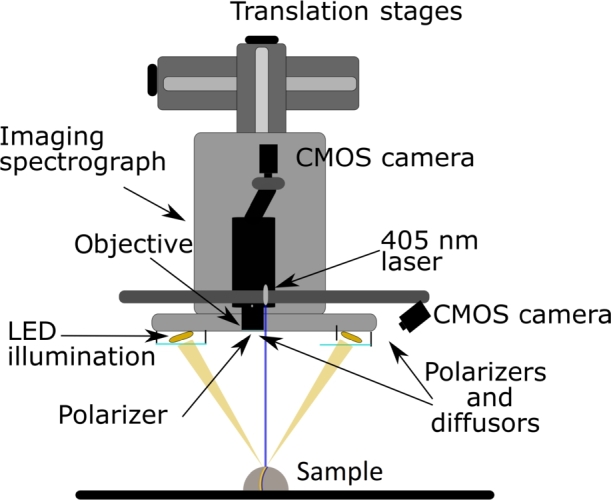
A schematic of the multimodal optical imaging system combining HSI and OP. Adapted from [42], [43].

This study utilized a custom-built integrated multimodal imaging system that combines HSI and 3D OP modules (Fig. 2) [40]. The hyperspectral imaging component of the system consists of several elements: an imaging spectrograph (ImSpector V10E, Specim, Finland), a monochrome CMOS camera (Blackfly S, BFS-U3-51S5M-C, FLIR, Canada), a 50 mm camera objective (Xenoplan 2.8/50-0902, Schneider-Kreuznach, Germany), a custom-built LED light source comprising four LED panels spanning the visible and near-infrared (NIR) ranges from 400 to 1000 nm, crossed polarizers (Bolder Vision Optik, Boulder, CO) to minimize specular reflection from the imaging sample, and two motorized translation stages (8MT195, Standa, Lithuania). The 3D OP module employs laser profilometry, which includes a laser (FLEXPOINT, 30 mW, 405 nm, LASER COMPONENTS, Germany) fixed parallel to the optical axis of the hyperspectral imaging module camera, a monochrome camera (Flea3, FL3-U3-13Y3M-C, FLIR, Canada), a 16 mm lens, and a 405 nm bandpass filter. This module relies on the laser line triangulation method, with a triangulation angle of 26° between the laser projector and the camera. The HSI module offers a spatial resolution of 100 μm in X and Y directions and a spectral resolution of 2.9 nm. The accuracy of the 3D surfaces captured by the OP module is 100 μm, 100 μm, and 50 μm in the X, Y, and Z directions, respectively.

The integration of the HSI and OP modules allows for the capture of the 3D surface shape of the imaged sample and enables the application of curvature and height corrections to the hyperspectral images [41]. This compensation addresses signal loss in hyperspectral images caused by high surface inclination angles or large distances, facilitating reliable image processing and analysis [42]. Multiple checkerboard measurements were conducted at various heights to ensure proper alignment of the two modules, resulting in a total image misalignment of less than 100 μm [42]. The hyperspectral imaging system was used in reflectance mode to acquire hyperspectral images of reflectance skin spectra of the biological samples. The exposure time for a single line acquisition was 250 ms, and the total acquisition time per hyperspectral image was about 3 minutes.

### 2.2 Animal experiments

The study involved six female BALB/c (BALB/cAnNCrl, Charles River Laboratories Italia s.r.l., Calco (Lecco), Italy) mice fourteen weeks old. Mice were housed in a specific pathogen-free environment with a 12-hour light-dark cycle at a temperature of 20–24 °C and relative humidity maintained at 55% ± 10%. They were provided *ad libitum* access to food and water.

A day before the start of the experiment, the backs of the mice were shaved and depilated using a depilatory cream (Reckitt, Slough, UK) to expose the bare skin and minimize light scattering caused by white hair. In some instances, depilation was repeated during the experiment due to the rapid regrowth of hair. The following day, tumors were induced by subcutaneous injection of $3\times 10^{5}$ CT26 murine colon carcinoma cells (American Type Culture Collection ATCC, Manassas, VA) in 100 μL of 0.9% NaCl saline onto the back of the mice. Before injection, the cells were cultured in Advanced RPMI 1640 (Gibco, Thermo Fisher Scientific, Waltham, MA) in a humidified incubator at 5% CO2 at 37 °C. Media were supplemented with GlutaMAX (100×, Gibco), 5% fetal bovine serum (FBS, Gibco), and Penicillin-Streptomycin (100×, Sigma-Aldrich, Merck, Darmstadt, Germany). The cells were routinely tested mycoplasma negative by MycoAlertTM PLUS Mycoplasma Detection Kit (Lonza, Basel, Switzerland).

**Figure 3:**
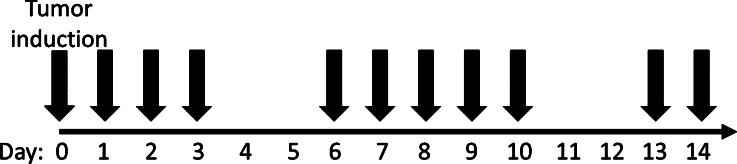
Timeline of the animal experiment. Black arrows represent the days when tumor imaging was performed. On Day 0, imaging was performed before and after tumor induction.

*In vivo* imaging of mice was conducted using the combined HSI and OP system at the Department of Experimental Oncology, Institute of Oncology Ljubljana, over 14 days. During the imaging sessions, the mice were anesthetized with 2% (v/v) isoflurane (Vetpharma Animal Health S.L., Barcelona, Spain) using VetFlo™ anesthesia system (Kent Scientific Corporation, Torrington, CT, USA). The experimental timeline involved the following steps (Fig. 3): a baseline image was recorded on the initial imaging day (Day 0), followed by subcutaneous injection of tumor cell suspension and the acquisition of another image post-injection. Subsequently, the growth of subcutaneously implanted tumors was monitored by capturing additional images on Days 1–3, 6–10, and 13–14.

### 2.3 Image preprocessing

To begin with, raw hyperspectral images were normalized using Eq. (1) to calculate actual tissue reflectance values ($I_{ref}$):

(1) $$I_{\mathrm{ref}}=\frac{I_{\mathrm{raw}}-I_{\mathrm{dark}}}{I_{\mathrm{white}}-I_{\mathrm{dark}}},$$

where $I_{raw}$ is raw hyperspectral image intensity, $I_{dark}$ is the dark current, and $I_{white}$ is white standard reference intensity (Spectralon, Labsphere Inc., North Sutton, NH) [7].

**Figure 4:**
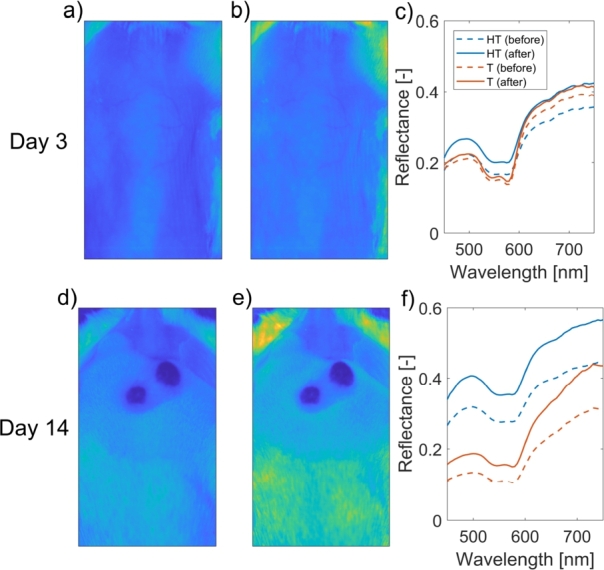
Curvature and height corrections: a) uncorrected 595 nm spectral band, b) corrected 595 nm spectral band, and c) corresponding average reflectance skin spectra of healthy tissue (HT) and tumor (T) before (dashed line) and after (solid line) corrections for subject 1 on Day 3. Plots d–f correspond to subject 1 on Day 14.

Subsequently, the 3D OP data were used for curvature and height corrections of hyperspectral images to mitigate the influence of tissue curvature, as described by Rogelj et al. [41], [42]. The results of the corrections are shown in Fig. 4(a–c) for subject 1 on Day 3 and in Fig. 4(d–f) for subject 1 on Day 14. Generally, the corrections affected healthy tissue spectra more than tumor spectra due to more pronounced curvature effects (a large area of healthy tissues on the backs and the sides of the mice). In this case, the large difference in magnitudes between the tumor and healthy tissue spectra on Day 14 is due to the high blood content in the tumor, which reduces its reflectance, and the high scattering in white hair, which increases the reflectance of healthy tissue. However, these differences arise from the underlying optical properties of tumors and healthy tissues and are not addressed nor compensated by height and curvature corrections. Moreover, the corrected hyperspectral images were spectrally reduced to a range of 450–750 nm with a spectral resolution of 5 nm to focus on the VIS spectral band. Additionally, they were spatially binned by a factor of two in both X and Y directions to facilitate subsequent image analysis. Thus, each preprocessed hyperspectral image was a data cube with a dimension of 612×400×61 pixels.

Then, the background not containing murine tissues was removed from hyperspectral images using the spectral angle mapper (SAM) method (Eq. (2)) by comparing the spectral similarity (angle) of pairs of measured reflectance spectra [7]:

(2) $$\theta =\arccos\left(\frac{\overset{\to }{s_{1}}\cdot \overset{\to }{s_{2}}}{\left|\left|\overset{\to }{s_{1}}\right|\right|\cdot \left|\left|\overset{\to }{s_{2}}\right|\right|}\right),$$

where $\overset{\to }{s_{1}}$ and $\overset{\to }{s_{2}}$ are corresponding spectra. $\theta =78.46^{\circ }$ was selected as the optimal threshold to segment tissues from the background.

**Figure 5:**
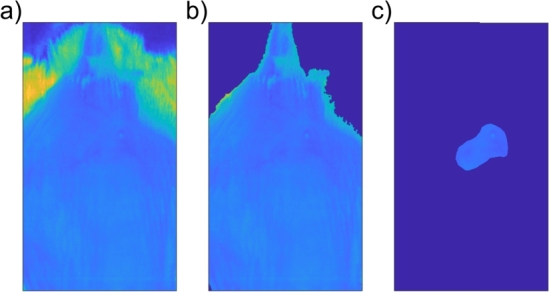
Background removal and tumor segmentation: a) corrected 595 nm spectral band, b) background removal using SAM, and c) manual tumor segmentation.

Finally, tumor segmentation was performed from hyperspectral images as follows: (1) erythema index (EI) [44], a ratio between the intensities of red and green components of reflected light, was calculated which provided the highest contrast of tumors; (2) smoothing and contrast enhancement of the EI images using *medfilt2* and *adapthisteq* functions from a standard image processing library in MATLAB R2022b (Mathworks, Natick, MA); and (3) manual segmentation of tumors from the processed EI images in Fiji 2.9.0 [45]. The manual segmentation was performed by an expert experimental oncologist involved in imaging and animal care. All other tissue was labeled as healthy tissue. The results of background removal using SAM and the manual segmentation of tumors are shown in Fig. 5 for subject 1 on Day 8.

### 2.4 Inverse adding-doubling algorithm

**Figure 6:**
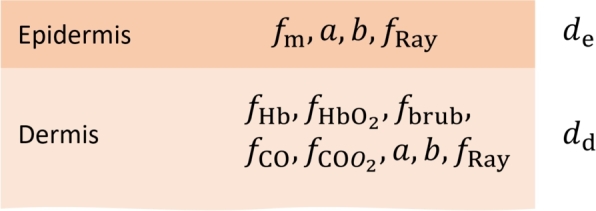
A two-layer murine skin model consisting of the epidermis and semi-infinite dermis with eleven tissue parameters: *f*_m_ – volume fraction of melanin; *f*_Hb_ – volume fraction of deoxyhemoglobin; $f_{{\mathrm{HbO}}_{2}}$ – volume fraction of oxyhemoglobin, *f*_brub_ – molar concentration of bilirubin; *f*_CO_ – molar concentration of reduced cytochrome C oxidase; $f_{{\mathrm{COO}}_{2}}$ – molar concentration of oxidized cytochrome C oxidase; *a* – scattering coefficient; *b* – scattering power; *f*_Ray_ – fraction of Rayleigh scattered light; *d*_e_ – epidermis thickness; *d*_d_ – dermis thickness. Parameters *a*, *b* and *f*_Ray_ are common for both layers. Adapted from [43], [47].

To extract information about tissue from normalized hyperspectral images containing reflectance skin spectra, an inverse adding-doubling (IAD) algorithm was developed in MATLAB R2022b (Mathworks, Natick, MA). IAD was accelerated by a graphics processing unit (GPU) to enable rapid and accurate simulation of light propagation in layered turbid media [46]. The accuracy and robustness of IAD for hyperspectral images were tested extensively and were previously reported, along with the specific details of the algorithm implementation and tissue modeling [47]. Briefly, a two-layer murine skin model (Fig. 6) consisting of an upper layer (epidermis) and a semi-infinite lower layer (dermis) was utilized, incorporating 11 model parameters that describe tissue physiology (e.g., melanin, hemoglobin) and morphology (e.g., scattering power). The absorption coefficients for the epidermis (Eq. (3)) and dermis (Eq. (4)) were calculated as follows [6]:

(3) $$\mu_{a,\;e}=f_{m}\mu_{a,\;m}+\mu_{a,\;\mathrm{base}},$$

(4) $$\mu_{a,\;d}=f_{\mathrm{Hb}}\mu_{a,\;\mathrm{Hb}}+f_{{\mathrm{HbO}}_{2}}\mu_{{a,\;\mathrm{HbO}}_{2}}+f_{\mathrm{brub}}\mu_{a,\;\mathrm{brub}}+f_{\mathrm{CO}}\mu_{a,\;\mathrm{CO}}+f_{{\mathrm{COO}}_{2}}\mu_{{a,\;\mathrm{COO}}_{2}}+\mu_{a,\;\mathrm{base}},$$

where $f_{m}$ is the volume fraction of melanin, $\mu_{a,m}$ is the melanin absorption coefficient, $f_{\mathrm{Hb}}$ and $f_{\mathrm{Hb}O_{2}}$ are volume fractions of deoxy- and oxyhemoglobin, $\mu_{a,\mathrm{Hb}}$ and $\mu_{a,\mathrm{Hb}O_{2}}$ are corresponding absorption coefficients and $f_{\mathrm{brub}}$ and $\mu_{a,\mathrm{brub}}$ are the molar concentration and absorption coefficient of bilirubin, respectively. Moreover, $f_{\mathrm{CO}}$ and $f_{\mathrm{CO}O_{2}}$ are molar concentrations of reduced and oxidized cytochrome C oxidase, whereas $\mu_{a,\mathrm{CO}}$ and $\mu_{a,\mathrm{CO}O_{2}}$ are associated absorption coefficients and $\mu_{a,\mathrm{base}}$ is the baseline absorption of bloodless skin. Ultimately, the reduced scattering coefficient (Eq. (5)) was defined as [6]:

(5) $$\mu_{s}^{\prime }=a[f_{\mathrm{Ray}}{\left(\frac{\lambda }{500\;\mathrm{nm}}\right)}^{-4}+(1-f_{\mathrm{Ray}}){\left(\frac{\lambda }{500\;\mathrm{nm}}\right)}^{-b}],$$

where *λ* is the wavelength of light, *a* is the reduced scattering coefficient at 500 nm, *b* is an exponential parameter related to the size of the Mie scatterers, and $f_{\mathrm{Ray}}$ represents the fraction of Rayleigh scattered light.

The measured reflectance spectra were fitted using the Levenberg-Marquardt (LM) algorithm adapted for GPU processing to extract all model parameters. Fitting was performed on a personal computer with an Nvidia Titan Xp graphics card with 12 GB RAM, AMD Ryzen 7 1700X processor, and 16 GB RAM.

### 2.5 Feature selection

After tissue properties were extracted using IAD from all hyperspectral images, total hemoglobin volume fraction, THB, and tissue oxygenation, ${\mathrm{StO}}_{2}$, were calculated using Eq. (6) and Eq. (7), respectively:

(6) $$\mathrm{THB}=100\cdot (f_{\mathrm{Hb}}+f_{\mathrm{Hb}O_{2}}),$$

(7) $${\mathrm{StO}}_{2}=100\cdot \frac{f_{\mathrm{Hb}O_{2}}}{f_{\mathrm{Hb}}+f_{\mathrm{Hb}O_{2}}}.$$

In total, ten tissue parameters were considered: $f_{m}$, $f_{\mathrm{Hb}}$, $f_{{\mathrm{HbO}}_{2}}$, THB, ${\mathrm{StO}}_{2}$, $f_{\mathrm{brub}}$, $f_{\mathrm{CO}}$, $f_{{\mathrm{COO}}_{2}}$, *a*, and *b*. These tissue properties are essential to discriminate tumors from healthy tissues since tumors can have altered physiology, metabolism, structure, and morphology, corresponding to light absorption and scattering changes [5], [11], [12].

For each tissue property, we calculated select first-order features: mean, minimum, maximum, standard deviation, skewness, kurtosis, entropy, and energy. Entropy and energy were calculated using Eq. (8) and Eq. (9), respectively [48], [49]:

(8) $$\mathrm{entropy}=-\sum_{i=1}^{N_{g}}p(i)\log_{2}(p(i)),$$

(9) $$\mathrm{energy}=\sum_{i=1}^{N_{p}}{\left(\text{X}(i)\right)}^{2},$$

where **X** is a set of $N_{p}$ pixels and p(i) is the normalized first order histogram with $N_{g}$ discrete intensity levels in the image. Moreover, we calculated the following Gray Level Co-occurrence Matrix (GLCM) features: contrast and homogeneity [50], [48], [49]. GLCM of size $N_{g}\times N_{g}$ represents the second-order joint probability distribution of pixel intensities within an image region defined by a mask, P(i,j|δ,θ) [48], [49]. The matrix element at position (i,j) denotes the frequency with which a pair of intensity levels, *i* and *j*, co-occur in two pixels that are separated by a distance of *δ* pixels at an angle *θ* [48], [49]. The distance *δ* from the center pixel or voxel is measured using the infinity norm. Specifically, contrast and homogeneity were calculated using Eq. (10) and Eq. (11), respectively [48], [49]:

(10) $$\mathrm{contrast}=\sum_{i=1}^{N_{g}}\sum_{j=1}^{N_{g}}{\left(i-j\right)}^{2}p(i,j),$$

(11) $$\mathrm{homogeneity}=\sum_{i=1}^{N_{g}}\sum_{j=1}^{N_{g}}\frac{p(i,j)}{1+|i-j|},$$

where p(i,j) is the normalized GLCM matrix. However, minimum and maximum values were excluded from the study because the boundaries in the IAD algorithm generally predetermined their values. In total, we had a set of 80 features (see Table A.1 in Appendix A) from which we determined the ten most important features using the Max-Relevance Min-Redundancy (MRMR) algorithm (Eq. (12)). The algorithm finds the most relevant features, *R*, with the least dependence, *D*, between the features:

(12) max⁡Φ(D,R)=D−R,

where Φ(D,R) is the operator combining relevance and dependence optimized in the process [51]. Feature selection generally improves the model interpretability and performance and reduces training time and memory burden.

Our data consisted of 11 hyperspectral images per mouse (one image per day) for all six mice included in the study, a total of 66 hyperspectral images. Hyperspectral images of two mice, 22 images in total, were set aside for validation, while the remaining 44 images were used for training and testing. We performed a 4-fold cross-validation, where image data of three mice was used to train the algorithms, and one was used for testing to avoid data contamination. In other words, since all features were calculated separately for tumors and healthy tissues, we had 126 data points for each feature, 66 of which were from healthy tissues and 60 from tumors, a well-balanced dataset. Eighty-four data points were used for training and testing, and 42 were set aside for validation.

### 2.6 Tissue classification

We utilized 30 supervised and unsupervised machine learning algorithms (see Table A.2 in Appendix A) from Python's *scikit-learn* toolbox to perform binary classification of tumors and healthy tissues based on the ten features determined by MRMR. Among the classifiers used were linear models, such as *LogisticRegression*, which is a fundamental linear classifier that predicts the probability of a certain class by fitting a logistic function to the data. It works by estimating the parameters (weights) that map input features to the target output, ensuring that the output lies between 0 and 1. Logistic regression is particularly useful for binary classification problems but can be extended to multi-class classification [52]. Moreover, *DecisionTreeClassifier* was used, which constructs a tree-like structure of decision rules derived from the input features. At each node, the algorithm selects the feature and threshold that result in the best split of the data. The resulting tree represents a series of decisions leading to a classification. Decision trees are intuitive and interpretable, making them valuable for understanding the decision-making process, but they are prone to overfitting, especially on noisy data [53]. Also, a Gaussian process classifier like *GaussianProcessClassifier* is a probabilistic model that leverages the power of Gaussian processes to perform non-linear classification tasks. It provides a flexible approach to modeling the underlying distribution of the data by assuming a prior distribution. The model computes the posterior distribution given the training data, making predictions based on this distribution. Gaussian process classifiers are powerful but computationally intensive [54]. Furthermore, ensemble-based methods were utilized, such as *AdaBoostClassifier*, which is a method that combines the predictions of several weak learners to form a strong classifier. The key idea is to sequentially train weak models and focus on the instances that previous models misclassified. This is done by adjusting the weights of misclassified instances, allowing subsequent models to focus more on the difficult cases. Such a model is robust to overfitting and can significantly improve the performance of simple models, but it may struggle with noisy data [55]. *ExtraTreesClassifier* is another ensemble method that builds a collection of decision trees. Unlike traditional decision tree models, each tree in the ensemble is built from the original data, and splits are made based on random thresholds. This approach increases the diversity among the trees and can lead to better generalization. The algorithm is less prone to overfitting compared to traditional decision trees and is computationally efficient [56]. In addition, various support vector machine (SVM) algorithms were employed. *SVC* is a versatile SVM implementation that supports both linear and non-linear classification. The core idea of SVM is to find the optimal hyperplane that maximizes the margin between different classes in the feature space. For non-linear problems, SVC uses kernel functions (e.g., polynomials) to map input data into higher-dimensional space, where a linear separator can be found. SVC is effective in high-dimensional spaces and is memory efficient [57]. *LinearSVC* is a specialized SVM that focuses on linear classification problems. It is well-suited for cases where the relationship between features and labels is approximately linear. It is particularly efficient for large datasets, as it implements the optimization algorithm in a way that scales better with the number of samples and features [57]. *NuSVC* is a type of standard SVC, which allows for finer control over the number of support vectors and the margin errors. It offers a more flexible way to balance the trade-off between the classifier's complexity and the margin size [58]. Lastly, neural networks (NN) like *MLPClassifier*, which is a type of feedforward artificial neural network that consists of multiple layers of nodes, where each node (except for the input nodes) represents a neuron that uses a non-linear activation function. It is a powerful model capable of learning complex non-linear relationships in data through backpropagation. It supports various configurations, such as the number of hidden layers, the number of neurons per layer, and different activation functions (e.g., ReLU, sigmoid) [59]. These algorithms collectively enabled a thorough exploration of classification performance across diverse modeling approaches. The list of all classifiers can also be seen in Fig. 13a and Fig. 14a. We employed different metrics to evaluate classifiers: accuracy, balanced accuracy, precision, recall, F1 score, and area under the curve (AUC) [60], [61].

**Figure 7:**
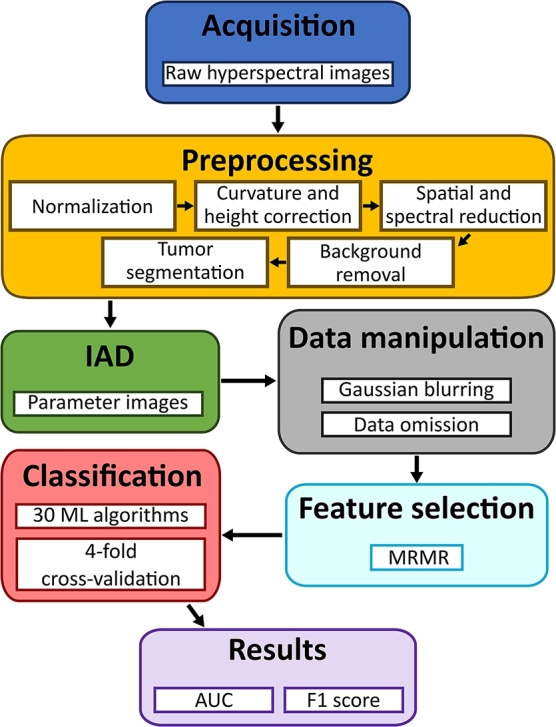
Schematic representation of the workflow.

For each of the 30 machine learning algorithms employed, we selected the best-performing model with the highest AUC based on the 4-fold cross-validation, as described in Section 2.5, and validated it on the validation set. Since our work focused on extracting accurate and robust biomarkers of a novel imaging technique, we performed two experiments to estimate the model performance in different scenarios. Firstly, we reduced the train and test sets by leaving out features from one day of animal experiments at a time for all days. Also, the data from the omitted day was not used for feature selection. Secondly, we smoothed the maps of tissue parameters extracted by IAD using a Gaussian filter with a smoothing kernel with a standard deviation of 0 to 10 with a step of 0.5. On both occasions, we repeated the feature selection using MRMR, model training, testing, and validation. We evaluated the robustness of all models on the validation set based on the AUC values and F1 scores. The schematic representation of our workflow is shown in Fig. 7.

## 3 Results

### 3.1 Tissue properties

**Figure 8:**
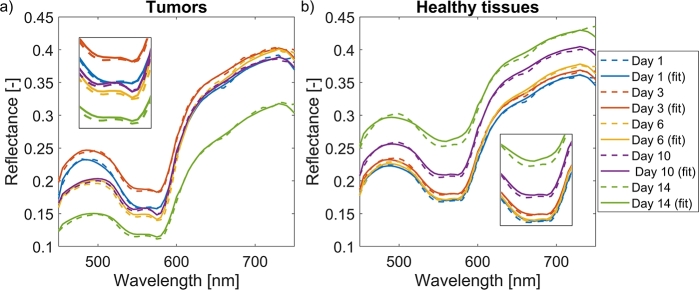
*In vivo* measured (dashed lines) and fitted (solid lines) reflectance skin spectra of mice on Days 1, 3, 6, 10, and 14: a) average spectra of all CT26 tumors, and b) average spectra of all healthy tissues.

After image preprocessing, hyperspectral images containing reflectance skin spectra were fitted using the IAD algorithm to extract model parameters (Fig. 6) describing murine skin. Fig. 8 shows the *in vivo* measured (dashed lines) and fitted (solid lines) reflectance skin spectra of tumors and healthy tissues. For both tissues, the average spectra for all subjects are presented on Days 1, 3, 6, 10, and 14. The fitted spectra matched closely with the measured spectra, with the most significant discrepancies being in the 550–600 nm spectral region and above 650 nm due to excess noise in the signal. However, our previous research on the accuracy and robustness of the IAD algorithm showed that the standard deviation of fitted spectra was within a few percent of the measured reflectance values [47].

More importantly, Fig. 8 shows the overall changes in tissue physiology. For tumors, we saw an initial increase in reflectance from Day 1 to Day 3, then a steady decrease until Day 6, followed by a significant drop on Day 14. Due to biological variability between different subjects, the standard deviations of reflectance spectra were generally between 2% and 3% and up to 5% for low reflectance values. We also noted a gradual increase in blood oxygenation until Day 10, as indicated by a pronounced camel hump in the 550–600 nm interval, followed by a slight decrease on Day 14. For healthy skin tissues, the changes in spectral shape were insignificant in the early days. However, we saw an increase in reflectance on Day 10 and especially Day 14. Also, healthy skin became less oxygenated as the camel hump was replaced by a single dip in the 550–600 nm spectral region (see inserts in Fig. 8).

**Figure 9:**
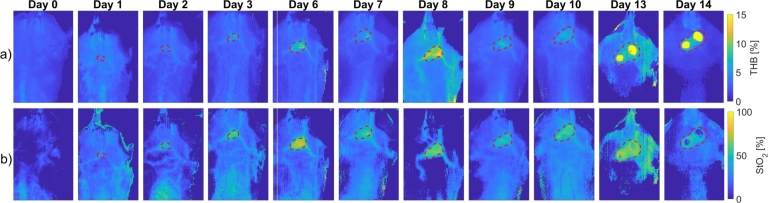
Colormaps of tissue properties for subject 1: a) total hemoglobin (THB) volume fraction, and b) tissue oxygenation (StO_2_). Dark colors represent low values, and light colors represent high values of THB and StO_2_. The background was removed from the images using the SAM method and appears dark on the colormaps. Tumors are outlined with red dashed lines according to the manual segmentations provided by a trained expert.

Fig. 9 shows colormaps of tissue properties extracted from hyperspectral images of subject 1 using the IAD algorithm. The top row (Fig. 9a) shows the total hemoglobin concentration (THB) evolution over 14 days. The tumor could be spotted in the upper central area of the image as early as Day 2 and became increasingly visible as the THB increased due to an increase in blood volume in the tumor. Meanwhile, Fig. 9b displays tissue oxygenation (${\mathrm{StO}}_{2}$), which also appears to have increased during the experiment for this subject.

**Figure 10:**
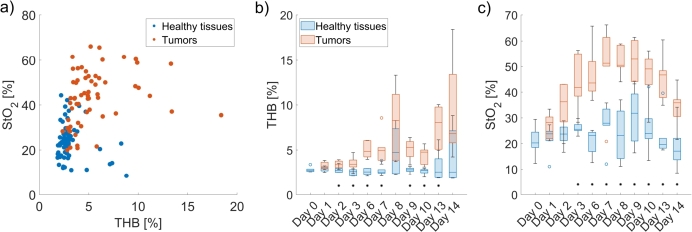
a) Scatter plot of mean THB and StO_2_ for all tumors and healthy tissues throughout all days of the experiment. Box plots of b) THB and c) StO_2_ for all CT26 tumors (in blue) and healthy tissues (in orange) during the experiment. Asterisk (*) denotes statistically significant differences (*p* < 0.05) between tumors and healthy tissues on a day-to-day basis.

Moreover, Fig. 10a shows the scatter plot of the mean THB and ${\mathrm{StO}}_{2}$ values across all days of the experiment. While the scatter plot demonstrates the imperfect separation between tumors and healthy tissues, it is crucial to consider the temporal aspect of our data. As our study involved longitudinal measurements, Fig. 10b,c shows the box charts of THB and ${\mathrm{StO}}_{2}$ for all subjects for each day during the experiment. THB for healthy tissues remained steady at around 2.7%±1.4%, while it slightly increased over time for tumors due to increased blood volume. (Fig. 10b). Similarly, ${\mathrm{StO}}_{2}$ for healthy tissues did not change substantially, while for tumors, it gradually increased from 28.2%±8.7% on Day 1 to 52.9%±13.8% on Day 9 and then gradually decreased in the follow-up to Day 14 (Fig. 10c). Notably, during the early days of the experiment, the THB and ${\mathrm{StO}}_{2}$ values for both tissues were less distinct, and the differences grew over time. By looking at day-to-day data, tumors could be discriminated from healthy tissues based on THB and ${\mathrm{StO}}_{2}$ as early as Day 2 and Day 3, respectively. Statistically significant differences (p<0.05) between tumors and healthy tissues on a day-to-day basis are denoted with an asterisk (*) in Fig. 10b,c.

Therefore, in the following two sections, we concentrated on identifying the most relevant features of CT26 tumors on a day-to-day basis and utilizing classic and advanced classifiers that can leverage temporal dynamics to discriminate them from healthy tissues.

### 3.2 Feature selection

We focused on establishing relevant indicators of CT26 tumor presence based on the features calculated for each tissue parameter image extracted using IAD from hyperspectral images.

**Figure 11:**
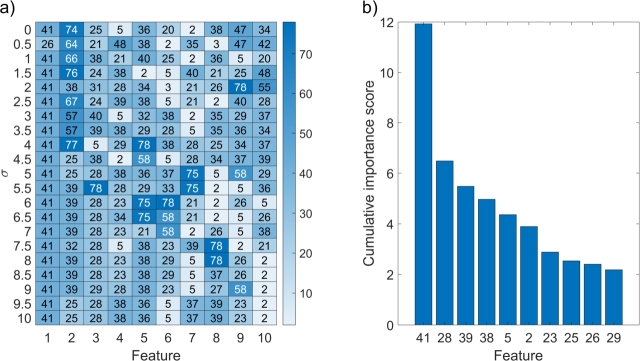
a) Heatmap of the ten most relevant features (x-axis) selected using the MRMR algorithm for different levels of Gaussian smoothing (y-axis). Selected feature 1 is the most relevant, and feature 10 is the least relevant of the selected ten features. Color coding provides the consecutive number of a feature from a pool of all features: features 1–10 are mean values of parameters, 11–20 standard deviations, 21–30 skewness, 31–40 kurtosis, 41–50 entropy, 51–60 contrast, 61–70 energy, and 71–80 homogeneity. b) Bar charts of cumulative importance scores calculated using MRMR for the ten most relevant features.

Fig. 11a shows the heatmap of the ten most relevant features selected with the MRMR algorithm for different levels of Gaussian blur applied to raw tissue parameter images. When no blur was applied, among the most relevant features were 41 (entropy of $f_{m}$), 74 (homogeneity of THB), 25 (skewness of ${\mathrm{StO}}_{2}$), and 5 (mean ${\mathrm{StO}}_{2}$). Except for feature 41, others are connected to blood concentration and oxygenation. As Gaussian smoothing with variable kernels was applied, the most relevant feature remained 41, followed by 28, 39, and 23 – the latter represent skewness of *a*, kurtosis of *b*, and skewness of *b*, respectively. These features are related to changes in scattering in tumors compared to healthy tissues. Shown in Fig. 11b are bar charts of cumulative importance scores for the ten most relevant features. We can see that the cumulative importance score of feature 41, the most important of all selected features, was almost 2x higher than the next most relevant feature. The importance scores for selected features decreased, and so did their variance.

**Figure 12:**
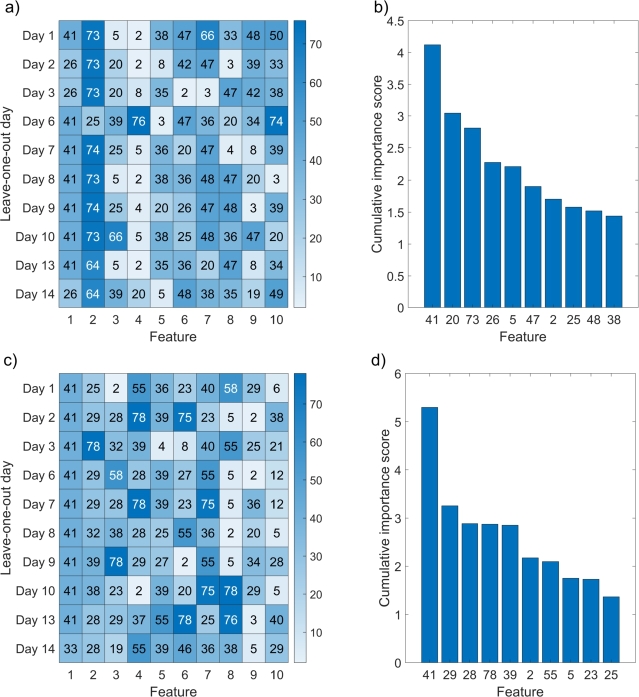
Heatmaps of the ten most relevant features selected using MRMR for different days left out of the feature selection (y-axis) for a) raw tissue parameter images (*σ* = 0) and c) tissue parameter images smoothed with a Gaussian kernel with *σ* = 5.0. Bar charts of cumulative importance scores for the ten most relevant features for b) raw tissue parameter images (*σ* = 0) and d) tissue parameter images smoothed with a Gaussian kernel with *σ* = 5.0.

Similarly, Fig. 12 shows the heatmaps of the ten most relevant features for a) raw and c) smoothed (σ=5.0) tissue parameter images when data from one of the experiment days was left out of feature selection, and model training and testing. Note that we did not leave out the data from Day 0 since the tumor cells were injected that day. Fig. 12b and Fig. 12d show that feature 41 was the most important in both cases, followed by 20 and 73 in the first and 29 and 28 in the second case. The former features are related to both scattering and blood content, while the latter are predominantly related to tissue scattering properties. We also noticed that the values of importance scores were less scattered for less relevant selected features in the case of Gaussian smoothing, as smoothing reduced the noise in the parameter images.

To sum up, the most important features were those related to $f_{m}$ entropy, skewness, and kurtosis of *a* and *b*, mean values of ${\mathrm{StO}}_{2}$ and $f_{\mathrm{Hb}}$, and standard deviations of $f_{{\mathrm{HbO}}_{2}}$ and ${\mathrm{StO}}_{2}$.

### 3.3 Tissue classification

**Figure 13:**
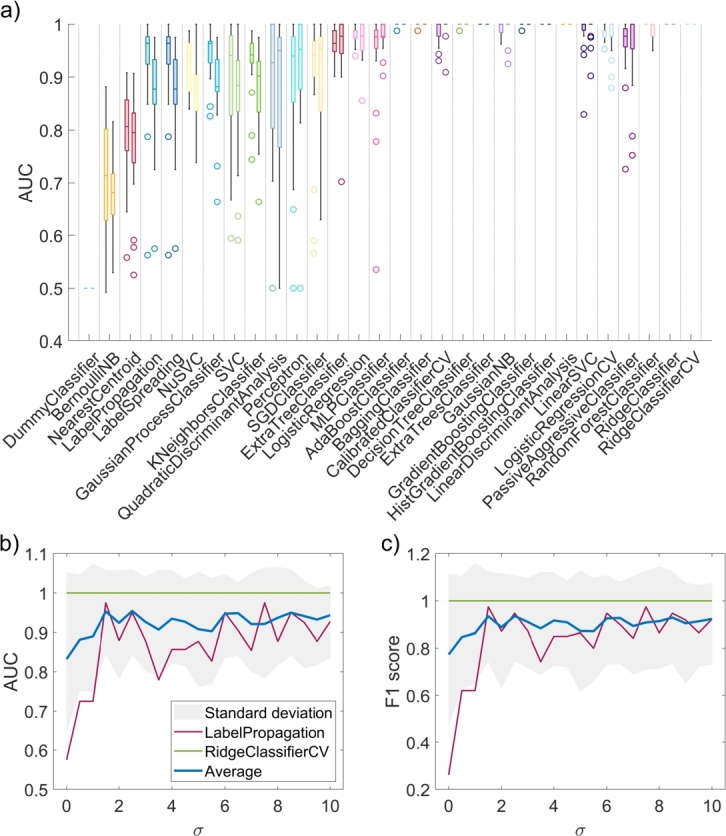
a) Box plots of AUC values on the validation set for 30 machine learning algorithms employed in this study for the first experiment, where tissue parameter images were smoothed with a variable Gaussian kernel. b) AUCs and c) F1 scores for a bad model (in red), best-performing model (in green), and average for all models (in blue) for a given Gaussian kernel size.

We classified the tissue using 30 standard ML algorithms following feature selection. Starting with the first experiment, where tissue parameter maps were smoothed with different Gaussian filters, Fig. 13a shows the AUC values for each model sorted by the ascending median value on the validation set. For each model, the box plots show AUC values for all Gaussian filters, and for each filter, ten features were selected, as seen in Fig. 11a. We can see that some algorithms, like *DummyClassifier*, consistently performed poorly, while algorithms like *BernoulliNB* performed better but were less robust. As the median AUC increased, the standard deviation was generally lower, resulting in high-performing models that were also very robust. Some algorithms, such as *RidgeClassifier*, consistently achieved an AUC of 1.

Moreover, shown in Fig. 13b and Fig. 13c are the AUC and F1 scores for one of the worst-performing models (in red), the best-performing model (in green), and the average scores for all models for a given Gaussian kernel (in blue). On average, initial tissue parameter smoothing improved the AUC and F1 scores by around 0.1. However, smoothing with a kernel greater than σ=1.5 did not improve the overall results, and error rates were relatively high for all kernel sizes but were the highest when no smoothing was applied (gray shaded area).

**Figure 14:**
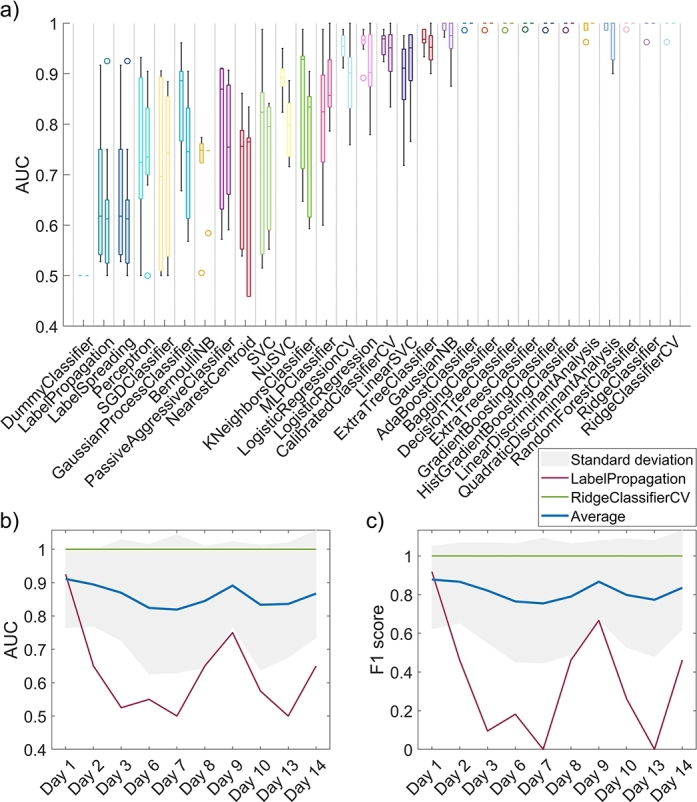
a) Box plots of AUC values on the validation set for 30 machine learning algorithms employed in this study for the second experiment, where training data was left out for one day at a time. b) AUCs and c) F1 scores for a bad model (in red), best-performing model (in green), and average for all models (in blue) for a given day omitted from the training process. All plots are for *σ* = 0.

As for the second experiment, where data from one day at a time was omitted from feature selection and training, we saw a similar trend with classifiers than previously. Shown in Fig. 14a are box plots of AUC values for each model for all omitted days. The ten most relevant features were selected for each omitted day, as seen in Fig. 12a,c based on the previous smoothing. Fig. 14a confirms that models based on random forests, gradient boosting methods, and linear discriminant analysis performed the best on our dataset, providing high prediction power and robustness.

From Fig. 14b and Fig. 14c, we can see that omitting early data on average improved the AUC and F1 scores since tumors could not be distinguished from healthy tissues based on physiological and morphological properties at early development stages. However, the omission of data on Days 6, 7, 10, and 13 reduced the overall performance of the models.

## 4 Discussion

The rapid development of optical imaging methods accelerates the ability to visualize and monitor tumors *in vivo* non-invasively, which has the potential to lead to the identification of innovative imaging biomarkers. These can significantly improve early cancer diagnosis and provide personalized therapeutic approaches. To make this possible, this work builds upon our previous efforts. In [40], we described the design, development, and validation of the combined HSI and 3D OP imaging system. In [41], [42], we thoroughly described the curvature and height corrections of hyperspectral images using profilometry data and showed they improved the parameter extraction from hyperspectral images. Then, in [47], we tested the robustness of the IAD algorithm for tissue parameter extraction using the two-layer skin model. Our proof-of-concept study [43] demonstrated that the combination of HSI and 3D OP could discriminate tumors from healthy tissues and that changes in growing CT26 tumors could be monitored daily using HSI [62]. Ultimately, in this work, we pave the way for discovering and validating hyperspectral imaging biomarkers for CT26 murine colon carcinomas using classic and advanced ML approaches that are clinically relevant yet interpretable.

The first part of our research focused on monitoring CT26 tumor progression continuously. Most previous studies were cross-sectional [19], [20], [21], [22], [23], [24], [26], [27], [28], [29], [31], [33], [34], where hyperspectral images were acquired at a single point in time, as opposed to our study and study of Sorg et al. [18], where longitudinal data was used. This is a significant advantage since we could detect day-to-day changes in tumor physiology and morphology and monitor the progression of the disease. It can be seen from Fig. 8a that spectral signatures of tumors were altered significantly as the tumors grew. An initial increase in reflectance from Day 1 to Day 3 was followed by a steady decrease until Day 6 and a significant drop on Day 14. This is due to a higher blood volume fraction in the tumor, and higher light absorption in the blood leads to reduced reflectance. Meanwhile, the changes in healthy tissues (Fig. 8b) were less noticeable in the first days of the experiment but were discernible later as the tumor progression affected the surrounding tissue. We saw an increase in reflectance on Day 10 and especially Day 14, probably because most blood supply was directed to tumors rather than neighboring skin. Fig. 9 shows the THB and ${\mathrm{StO}}_{2}$ parameter images from the first to the last day of the animal experiment. The daily changes visible by the naked eye were confirmed in Fig. 10, which shows THB gradually increased over time for tumors due to the increased blood volume, while ${\mathrm{StO}}_{2}$ increased twofold midway through the experiment and then gradually decreased. The observed increase in ${\mathrm{StO}}_{2}$ could be attributed to the formation and expansion of new blood vessels that supply a large amount of oxygenated blood to the tumor to meet the increased demand. However, the tumor began to outgrow the capacity of its existing vasculature, leading to a gradual decrease in oxygen saturation due to insufficient oxygen supply. On the other hand, the changes in healthy tissues were less pronounced and could be attributed to day-to-day variations in mice positioning during imaging and to repeated depilation on Day 9 for selected subjects.

Moreover, we concentrated on selecting relevant features describing the physiology and morphology of tumors and healthy tissues. Most other studies used spectral signatures (e.g., reflectance skin spectra or their principal components) as input [20], [21], [22], [23], [24], [25], [26], [27], [28], [29], [31], [33], [34], while we extracted tissue properties related to tumor biology to improve interpretability and consider clinical relevance to aid in disease diagnosis. We also identified the most robust and stable features by heavily testing the MRMR algorithm for feature selection based on two scenarios: 1) parameter images smoothing with different Gaussian kernels and 2) omitting training data of each day of the animal experiment at a time. Ultimately, we utilized classic and advanced ML techniques to discriminate tumors from healthy tissues and tested the reliability of models for the two scenarios.

In the first experiment, feature 41 ($f_{m}$ entropy) was the most relevant to distinguishing CT26 colon carcinomas from healthy tissues, as seen in Fig. 11. Although CT26 tumor models do not produce melanin physiologically, we have found a strong interplay between $f_{m}$ and necrosis, which results in a significant difference in melanin entropy between tumors and healthy tissues. The entropy was lower in tumors than in healthy tissues, suggesting that the spatial distribution of melanin in CT26 tumor models is homogeneous and orderly. Other relevant features were related to scattering (*a* and *b*), which highlights the changes in tumor morphology that could be related to the infiltration of immune cells and changes in tissues due to the edema. Lastly, some prominent features were related to blood content (THB and ${\mathrm{StO}}_{2}$), which corresponds to blood volume and oxygenation alterations, as there was generally more blood and higher oxygenation in CT26 tumors than in healthy tissues. Finally, we showed that variable Gaussian smoothing improved the performance of ML models on both training and validation sets by up to 0.1 for both AUC and F1 scores (Fig. 13b,c), as most noise was removed from the images. However, the improvement was only noticeable up to σ=1.5. Evaluating the model's performance on both training and validation sets helped us ensure we avoided potential issues, such as under- and overfitting. High accuracy on the training set and low accuracy on the validation set imply overfitting, leading to low generalizability. In contrast, low accuracy in both sets would suggest that the model could not capture the complexity of the data and is thus underfitting.

Similar findings were observed in the second experiment. Feature 41 was the most relevant in all cases, but its cumulative importance score was slightly less outstanding than in the first experiment. As training data from different days of the experiment was omitted, various features stood out, as seen in Fig. 12. When no smoothing was applied (σ=0), features related to blood content were much more important for classification than with smoothing (σ=5.0) applied. We can explain this by much higher variation in parameters related to blood, also leading to more considerable differences between tumors and healthy tissues. As parameter images were smoothed, the variation and, thus, differences between tissues became less prominent, resulting in lower predicting power. The results in Fig. 14 indicate that early-stage data was not as informative for distinguishing between CT26 tumors and healthy tissues based on physiological and morphological properties. The most significant improvement could be achieved by omitting Day 9 because three mice were re-depilated at that time due to excess hair growth, causing erythema that overshadowed other differences in healthy tissues and tumors. On the other hand, data from Days 6, 7, 10, and 13 played a crucial role in achieving accurate classification results because its omission resulted in the most considerable decrease in overall model performance. These findings underscore the importance of careful data curation and understanding the underlying biological or experimental factors that could impact model performance in medical classification tasks.

All in all, the most important features were those related to $f_{m}$ entropy, skewness, and kurtosis of *a* and *b*, mean values of ${\mathrm{StO}}_{2}$ and $f_{\mathrm{Hb}}$, and standard deviations of $f_{{\mathrm{HbO}}_{2}}$ and ${\mathrm{StO}}_{2}$. Among the best-performing models were *AdaBoostClassifier*, *ExtraTreesClassifier*, *GradientBoostingClassifier*, and *RidgeClassifier*.

Testing a wide range of 30 ML algorithms from Python's *scikit-learn* toolbox allowed us to conduct a thorough benchmark, ensuring that our approach to identifying hyperspectral imaging biomarkers of CT26 tumors is reliable and robust. This comprehensive evaluation provided valuable insights into how different algorithms handle the complexities of distinguishing between CT26 tumors and healthy tissues, given the unique characteristics of our dataset. Each algorithm was assessed using default *scikit-learn* hyperparameter settings. This approach enabled us to understand the varying sensitivities of different algorithms to data characteristics such as distribution, noise, and feature interactions. By evaluating a broad spectrum of algorithms, we identified those that demonstrated the most resilience and consistency, thus ensuring the robustness of our results and avoiding biases that might have arisen from focusing on a limited set of algorithms or different fine-tuning techniques.

Our study is limited by a relatively small dataset (84 data points for training and testing and 42 for validation) due to ethical guidelines for reducing animals in animal experiments. Therefore, we carefully split data and performed 4-fold cross-validation on data points from four mice, where three mice were used for training and one for testing to avoid data contamination. However, our data for consecutive days for each mouse was still somewhat similar, especially in the early days when no significant alterations happened in tumors. The small dataset also did not allow us to utilize state-of-the-art deep learning methods to perform tasks such as tumor segmentation.

Although we evaluated a wide range of machine learning algorithms, we relied on default hyperparameter settings without fine-tuning the models. Hyperparameter tuning is a crucial step in machine learning, as default values are merely a starting point and may lead to suboptimal performance on specific datasets. By fine-tuning the hyperparameters, it is likely that the performance of these models could be significantly enhanced. We recognize that many of the algorithms we employed could be improved through fine-tuning and that the absence of this step might have led to some models performing sub-optimally, potentially affecting our selection of the best-performing algorithms.

Our proposed approach has demonstrated potential in analyzing tumor progression, but several factors could impact the accuracy and reliability of the imaging results and subsequent tumor analysis. These factors may have contributed to the high THB value observed on Day 8, seen in Fig. 9. One potential issue is the inconsistent positioning of the mice during different imaging sessions, which can lead to misalignment of the images. Such misalignment introduces artifacts or errors due to varying degrees of tissue compression or distortion, causing variations in THB values and other tissue parameters across sessions. Another factor is hair growth in the mice over time, which can lead to increased light scattering and reflectance during imaging. This can introduce noise and artifacts in the hyperspectral images, potentially affecting the accurate estimation of tissue parameters such as THB. The impact of hair growth may become more pronounced in later imaging sessions. Additionally, incomplete thermalization of the LED illumination used for hyperspectral imaging can cause fluctuations in the captured spectra if the LEDs do not reach thermal equilibrium before image acquisition. These fluctuations can ultimately lead to inconsistencies in tissue parameter values. Moreover, height and curvature corrections applied during the imaging process altered the shapes of the reflectance skin spectra to some degree. Furthermore, the IAD algorithm used to fit reflectance spectra to extract tissue parameters may occasionally converge to a local minimum instead of the global minimum, leading to sub-optimal fitting results. Such deviations could cause variations in the estimated parameter values, such as THB, particularly in cases where the algorithm is sensitive to initial conditions or noise in the data. All these factors could ultimately affect the estimation of tissue parameters by the IAD algorithm, which could, in turn, impact the discrimination of CT26 tumors from healthy tissues using the proposed machine learning models.

Our primary focus in the future will be expanding our dataset with other mouse types, such as hairless mice, and other murine tumor models, such as melanoma, mammary carcinoma, mammary adenocarcinoma, and oral carcinoma. We will establish clinical tumor models based on preclinical tumor models to help clinicians provide a more precise diagnosis, leading to appropriate treatment strategies and improved treatment outcomes. An extensive and diverse dataset will allow us to explore further the impact of our data and the IAD algorithm itself on feature selection and model prediction, such as the interplay between necrosis and $f_{m}$. Future work will also involve fine-tuning to optimize the performance of the most promising algorithms identified in this study, as this will help tailor the models more precisely to our dataset and enhance their predictive accuracy.

Lastly, we can utilize advanced deep learning methods for different purposes. Deep learning algorithms, particularly CNNs, could automatically extract intricate features from hyperspectral images to improve the classification at the expense of interpretability. Also, we could develop deep learning models for tumor segmentation based on the features extracted from hyperspectral images and compare their performance with the manual segmentations from trained experts. Another possibility would be to compare the performance of classification and segmentation models to study the trade-off between the accuracy of overall image labeling and detailed pixel-level labeling. Moreover, we will work on incorporating multimodal data from the 3D OP module built into the HSI system, enhancing the accuracy of tumor classification by considering a more comprehensive set of morphological, structural, and volumetric features.

Importantly, deep learning will allow us to process images in real-time, allowing for rapid assessment of tissues in a clinical setting. In our recent work, we have shown that advanced deep learning models could expedite the extraction of tissue parameters from hyperspectral images to process about two images per second [63], which is suitable for clinical use. However, the current experimental setup does not allow real-time image acquisition and preprocessing. While image analysis (parameter extraction) is the most time-consuming task, the experimental system must be upgraded before real-time assessment of tissues in clinical settings is possible.

## 5 Conclusion

In conclusion, we presented a novel non-invasive tumor growth monitoring approach based on hyperspectral imaging and optical profilometry. Our study demonstrates the feasibility of contactless and non-invasive skin and subcutaneous tumor detection by harnessing visible and near-infrared light for inherent tissue contrast.

By leveraging various ML algorithms, we achieved high discrimination accuracy (AUC and F1 score of up to 1) and showcased the potential for enhancing diagnostic insights into tumor microenvironments. Furthermore, we addressed the robustness of our approach: smoothing tissue parameter maps with different Gaussian kernels improved the average AUC by 0.1; omitting training data on Days 1–3 and Day 9 of the experiment led to notable improvements in model performance.

Our findings underscore the significance of combining innovative optical imaging techniques with cutting-edge machine learning approaches, paving the way for precise and robust imaging biomarkers that could aid tumor diagnosis and ultimately advance clinical practice.

## Ethics statement

Approval of all ethical and experimental procedures and protocols in animals was granted by the Ministry of Agriculture, Forestry and Food of the Republic of Slovenia (permission no. U34401-3/2022/11). The experimental procedures complied with the guidelines for animal experiments of the EU directive (2010/63/EU) and ARRIVE guidelines.

## Funding

This work was supported by Slovenian Research and Innovation Agency (10.13039/501100004329ARIS) grants P1-0389, P3-0003, Z1-4384, J3-2529, and J3-3083.

## CRediT authorship contribution statement

**Tadej Tomanic:** Writing – review & editing, Writing – original draft, Visualization, Software, Methodology, Investigation, Formal analysis, Data curation. **Jost Stergar:** Writing – review & editing, Supervision, Resources, Methodology, Funding acquisition, Data curation, Conceptualization. **Tim Bozic:** Writing – review & editing, Validation, Methodology, Investigation. **Bostjan Markelc:** Writing – review & editing, Validation, Supervision, Resources, Project administration, Methodology, Investigation, Funding acquisition, Conceptualization. **Simona Kranjc Brezar:** Writing – review & editing, Validation, Resources, Methodology, Investigation, Conceptualization. **Gregor Sersa:** Writing – review & editing, Validation, Supervision, Resources, Project administration, Funding acquisition, Conceptualization. **Matija Milanic:** Writing – review & editing, Validation, Supervision, Resources, Project administration, Methodology, Funding acquisition, Data curation, Conceptualization.

## Declaration of Competing Interest

The authors declare that they have no known competing financial interests or personal relationships that could have appeared to influence the work reported in this paper.

## Data Availability

The code, data, and materials that support the findings of this study are available from the corresponding author upon reasonable request.

## Appendix Supplementary material

MMC Supplementary materials (Table A.1 and A.2) are available online as supporting information. MMC
