# Supplementary material for: Towards reliable hyperspectral imaging biomarkers of CT26 murine tumor model
Source: Heliyon. 2024 Oct 26;10(21):e39816. doi: 10.1016/j.heliyon.2024.e39816 (PMC11567117; doi:10.1016/j.heliyon.2024.e39816)
Supplement: MMC — Supplementary materials (Table A.1 and A.2) are available online as supporting information. [file mmc1.pdf]

Towards Reliable Hyperspectral Imaging Biomarkers of CT26 Murine Tumor Model

Appendix A: Supplementary materials

Table A.1: A full set of features used in the study.

No.	Name	No.	Name
1	Mean f_{m}	41	Entropy of f_{m}
2	Mean f_{Hb}	42	Entropy of f_{Hb}
3	Mean $f_{\mathrm{HbO_2}}$	43	Entropy of $f_{\mathrm{HbO_2}}$
4	Mean $\mathrm{StO_2}$	44	Entropy of $\mathrm{StO_2}$
5	Mean THB	45	Entropy of THB
6	Mean f_{brub}	46	Entropy of f_{brub}
7	Mean f_{CO}	47	Entropy of f_{CO}
8	Mean $f_{\mathrm{COO_2}}$	48	Entropy of $f_{\mathrm{COO_2}}$
9	Mean a	49	Entropy of a
10	Mean b	50	Entropy of b
11	Standard deviation of f_{m}	51	Contrast of f_{m}
12	Standard deviation of f_{Hb}	52	Contrast of f_{Hb}
13	Standard deviation of $f_{\mathrm{HbO_2}}$	53	Contrast of $f_{\mathrm{HbO_2}}$
14	Standard deviation of $\mathrm{StO_2}$	54	Contrast of $\mathrm{StO_2}$
15	Standard deviation of THB	55	Contrast of THB
16	Standard deviation of f_{brub}	56	Contrast of f_{brub}
17	Standard deviation of f_{CO}	57	Contrast of f_{CO}
18	Standard deviation of $f_{\mathrm{COO_2}}$	58	Contrast of $f_{\mathrm{COO_2}}$
19	Standard deviation of a	59	Contrast of a
20	Standard deviation of b	60	Contrast of b
21	Skewness of f_{m}	61	Energy of f_{m}
22	Skewness of f_{Hb}	62	Energy of f_{Hb}
23	Skewness of $f_{\mathrm{HbO_2}}$	63	Energy of $f_{\mathrm{HbO_2}}$
24	Skewness of $\mathrm{StO_2}$	64	Energy of $\mathrm{StO_2}$
25	Skewness of THB	65	Energy of THB
26	Skewness of f_{brub}	66	Energy of f_{brub}
27	Skewness of f_{CO}	67	Energy of f_{CO}
28	Skewness of $f_{\mathrm{COO_2}}$	68	Energy of $f_{\mathrm{COO_2}}$
29	Skewness of a	69	Energy of a
30	Skewness of b	70	Energy of b
31	Kurtosis of f_{m}	71	Homogeneity of f_{m}
32	Kurtosis of f_{Hb}	72	Homogeneity of f_{Hb}
33	Kurtosis of $f_{\mathrm{HbO_2}}$	73	Homogeneity of $f_{\mathrm{HbO_2}}$
34	Kurtosis of $\mathrm{StO_2}$	74	Homogeneity of $\mathrm{StO_2}$
35	Kurtosis of THB	75	Homogeneity of THB
36	Kurtosis of f_{brub}	76	Homogeneity of f_{brub}
37	Kurtosis of f_{CO}	77	Homogeneity of f_{CO}
38	Kurtosis of $f_{\mathrm{COO_2}}$	78	Homogeneity of $f_{\mathrm{COO_2}}$
39	Kurtosis of a	79	Homogeneity of a
40	Kurtosis of b	80	Homogeneity of b

Table A.2: Machine learning algorithms employed in the study.

No.	Name	No.	Name
1	*AdaBoostClassifier*	16	*LinearDiscriminantAnalysis*
2	*BaggingClassifier*	17	*LinearSVC*
3	*BernoulliNB*	18	*LogisticRegression*
4	*CalibratedClassifierCV*	19	*LogisticRegressionCV*
5	*DecisionTreeClassifier*	20	*MLPClassifier*
6	*DummyClassifier*	21	*NearestCentroid*
7	*ExtraTreeClassifier*	22	*NuSVC*
8	*ExtraTreesClassifier*	23	*PassiveAggressiveClassifier*
9	*GaussianNB*	24	*Perceptron*
10	*GaussianProcessClassifier*	25	*QuadraticDiscriminantAnalysis*
11	*GradientBoostingClassifier*	26	*RandomForestClassifier*
12	*HistGradientBoostingClassifier*	27	*RidgeClassifier*
13	*KNeighborsClassifier*	28	*RidgeClassifierCV*
14	*LabelPropagation*	29	*SGDClassifier*
15	*LabelSpreading*	30	*SVC*
